# Diagnostic Performance and Prognostic Value of Serum Amyloid A in Patients with Bloodstream Infection

**DOI:** 10.3390/diagnostics16101510

**Published:** 2026-05-16

**Authors:** Hyein Kang, Sunggyun Park

**Affiliations:** Department of Laboratory Medicine, Keimyung University School of Medicine, Daegu 42601, Republic of Korea; graceinkang@dsmc.or.kr

**Keywords:** serum amyloid A (SAA), bloodstream infection, acute-phase reactants, inflammation, diagnostic markers

## Abstract

**Background:** Serum amyloid A (SAA) is an acute-phase reactant that increases rapidly in response to inflammatory stimuli and infection, earlier and more markedly than conventional markers such as C-reactive protein (CRP). However, large-scale evidence of its clinical utility in bloodstream infections (BSIs) remains limited. This study aimed to evaluate the diagnostic and prognostic value of SAA in patients presenting to the emergency department (ED) with suspected infection. **Methods:** We retrospectively reviewed the electronic medical records of adult patients who underwent simultaneous SAA and blood culture (BC) testing at the ED of a tertiary referral hospital between January and December 2025. Initial laboratory data, including CRP, procalcitonin (PCT), white blood cell (WBC) count, and absolute neutrophil count (ANC), were collected, and BSI was defined as the isolation of pathogenic organisms in BC. Correlations and agreement between SAA and other markers were assessed, and the diagnostic performance of BSI was evaluated using receiver operating characteristic curves and the area under the curve (AUC). Survival outcomes were analyzed using the Kaplan–Meier method. **Results:** Among the 3321 included patients, 379 patients (11.4%) had positive BCs. Median SAA levels were significantly higher in BSI patients than in non-BSI patients (202.0 vs. 71.0 mg/L, *p* < 0.001), with the highest levels observed in Gram-negative infections. SAA showed a strong correlation with CRP (*r_s_* = 0.884) and a moderate correlation with PCT (*r_s_* = 0.576). The AUC for BSI diagnosis was highest for PCT (0.789), followed by SAA (0.650). The SAA demonstrated high sensitivity (90.5%) but low specificity (26.9%). Higher SAA levels were significantly associated with increased mortality rates. **Conclusions:** In adult ED patients, SAA is significantly associated with BSI and mortality and is a sensitive biomarker for the early detection of BSIs. Although SAA alone showed inferior discriminative performance compared to PCT, it may serve as an adjunctive screening tool in the ED, particularly in settings where PCT availability is limited.

## 1. Introduction

Acute-phase reactants (APRs) are proteins whose concentrations increase or decrease by at least 25 percent during inflammation, infection, trauma, surgery, burns, or other events [1,2,3]. The serum amyloid A (SAA) family, known to contain a number of differentially expressed apolipoproteins synthesized primarily by the liver, responds rapidly to proinflammatory signals such as cytokines like interleukin (IL)-6, IL-1, and TNF-α [1,4]. SAA is present at blood concentrations below 3 mg/L in healthy individuals; however, it increases up to 1000-fold within 24 h after stimulation by proinflammatory cytokines [4]. C-reactive protein (CRP) is the most widely used APR in clinical practice and has been extensively characterized across a broad spectrum of diseases [5]. However, compared to CRP, SAA begins to increase approximately 8 h after the onset of the acute-phase response and reaches a peak at 24 to 48 h, demonstrating a more rapid response than CRP [6]. This property may be advantageous in the early detection and monitoring of acute inflammatory conditions. Procalcitonin (PCT), another APR that has been investigated relatively recently, has been shown to guide antibiotic therapy in critically ill patients with bacterial infections [7,8]. In addition to CRP and PCT, various other APRs and infection-related biomarkers have been investigated, including haptoglobin, ceruloplasmin, fibrinogen, and erythrocyte sedimentation rate (ESR), as well as more recently introduced markers such as presepsin [9,10]. Simultaneous measurement of these markers may provide complementary information and facilitate a more comprehensive assessment of the infection burden, systemic inflammatory response, and prognosis in acutely ill patients [5,11,12,13].

Several studies have evaluated the diagnostic utility of SAA in diverse disease contexts, including neonatal and adult sepsis, acute appendicitis, rheumatic diseases, inflammatory bowel disease, ankylosing spondylitis, and human immunodeficiency virus (HIV)-associated pulmonary infections [4,14,15,16,17,18,19,20]. Despite these accumulating studies, large-scale studies specifically assessing the diagnostic value of SAA in patients with bloodstream infections (BSIs) presenting to the emergency department (ED) remain limited. BSIs are associated with substantial morbidity and mortality, and early recognition is essential for the prompt initiation of appropriate antimicrobial therapy and supportive care [21]. Blood culture (BC) remains the reference standard for the diagnosis of BSI, but it requires at least 24 to 48 h before pathogen identification and susceptibility testing become available [22,23]. Therefore, reliable blood-based biomarkers that can assist clinicians in identifying patients at high risk of BSI at the time of ED presentation are of great clinical interest.

In this context, we aimed to analyze the correlation between SAA and other commonly used inflammatory markers, including CRP, PCT and absolute neutrophil count (ANC), in adult patients who presented to the ED of a tertiary referral hospital and underwent SAA measurement and BC testing simultaneously. By focusing on a large real-world cohort spanning an entire year, our study sought to capture the full spectrum of disease severity encountered in the ED. In addition, we sought to evaluate the diagnostic performance of SAA for BSIs, compare its area under the curve (AUC) and optimal cutoff value with those of conventional markers, and examine whether SAA could serve as a useful predictor of clinical outcomes, including in-hospital mortality and short-term survival. Finally, by stratifying BC-positive patients according to pathogen category (Gram-positive [GP] bacteria, Gram-negative [GN] bacteria, and fungi), we explored potential differences in the SAA response between distinct microbiological etiologies, which may shed light on the underlying immunopathological mechanisms of SAA induction in BSI.

## 2. Materials and Methods

### 2.1. Study Population

Electronic medical records of patients with SAA and blood culture results who visited the ED of Keimyung University Dongsan Hospital, a tertiary referral hospital in Daegu, Korea, from January to December 2025 were reviewed retrospectively. Demographic characteristics, including age and sex, were collected for all patients. The initial laboratory parameters measured at the time of ED presentation, including SAA, CRP, PCT, complete blood count (CBC) with white blood cell (WBC) differential counts, and blood culture results, were retrieved. BSI was defined as the identification of pathogenic organisms in at least one BC set, excluding episodes classified as contamination as described above. Positivity was assessed based on the results from this initial collection; patients with subsequent blood cultures were not included in the positivity assessment for this study. Mortality status during the index hospitalization was assessed using hospital administrative and clinical records, and the time interval from ED presentation to death was calculated for non-survivors. Patients younger than 19 years of age and those with BC contamination were excluded from the study according to predefined criteria. Blood culture contamination was defined as the isolation of organisms such as *Bacillus* spp., *Corynebacterium* spp., *Propionibacterium acnes*, and coagulase-negative *Staphylococci* in only one bottle of a blood culture set, following established contamination criteria [24].

This study was approved by the Institutional Review Board (IRB) of Keimyung University Dongsan Hospital, Daegu, Korea (IRB No. DSMC 2026-03-027). Given the retrospective design and the use of de-identified data, the requirement for informed consent was waived in accordance with national regulations and institutional policies.

### 2.2. Assay Methods

Serum SAA and CRP levels were analyzed using a c702 chemistry analyzer (Roche Diagnostics, Mannheim, Germany), which is an automated platform utilizing immunoturbidimetric assays. PCT levels were analyzed using an e801 immunoassay analyzer (Roche Diagnostics), which utilizes the electrochemiluminescence immunoassay method. CBC and WBC differential counts were performed using an automated hematology analyzer, XN-1000 (Sysmex, Kobe, Japan). For BCs, venous samples were collected aseptically and inoculated into two sets of aerobic and anaerobic BC bottles (BacT/ALERT FA/FN Plus, bioMérieux, Marcy l’Étoile, France). The bottles were incubated and monitored by an automated BC system. When growth was detected, microorganisms were identified by VITEK MS (bioMérieux), a matrix-assisted laser desorption/ionization time-of-flight mass spectrometry (MALDI-TOF-MS) system. All laboratory tests were performed according to the manufacturer’s instructions and internal laboratory standard operating procedures. For SAA, CRP, and PCT, results below the lower limit of detection or above the upper limit of quantification were assigned the lower or upper limit values, respectively, to enable uniform statistical analysis. The measured concentrations and conventional cutoffs at our institution were as follows: SAA, 5.0–2400 mg/L (cutoff: 10.0 mg/L); CRP, 0.03–70 mg/dL (cutoff: 0.5 mg/dL); and PCT, 0.02–100 ng/mL (cutoff: 0.046 ng/mL). For the agreement and survival analyses, test positivity was defined according to these cutoff values.

### 2.3. Statistical Analysis

Continuous variables were expressed as the median and interquartile range (IQR) due to the skewed distributions of inflammatory biomarker levels. Comparisons between two groups (e.g., BC-positive vs. BC-negative, survivors vs. non-survivors) were performed using the Mann–Whitney U test, whereas comparisons across three groups (e.g., GP vs. GN vs. fungal BSIs) were conducted using the Kruskal–Wallis test, followed by appropriate post hoc pairwise analyses with *p*-value adjustment when applicable.

Agreement between binary test results (positive vs. negative) was evaluated using Cohen’s kappa coefficient, with test results classified as positive or negative based on the predefined cutoff values for SAA, CRP, and PCT. Positive and negative agreement rates with 95% confidence intervals (CIs) were also calculated.

Diagnostic performance for BSI was evaluated by calculating sensitivity, specificity, and their 95% CIs. Receiver operating characteristic (ROC) curves were constructed for each biomarker, and the AUCs were reported. For combined markers, logistic regression models were used to generate predicted probabilities, from which ROC curves and AUCs were derived. The optimal cutoff for SAA in predicting BSI was determined by maximizing Youden’s index, with corresponding odds ratios (ORs) calculated.

Correlation analyses between SAA and other inflammatory markers were performed using Spearman’s rank correlation coefficient (Spearman’s *r_s_*), and the corresponding 95% CIs were estimated; *p* < 0.05 was considered statistically significant.

Survival analysis was performed using the Kaplan–Meier method, with patients stratified into positive or negative groups according to the cutoff values. The association between SAA levels and the length of hospital stay among non-survivors was explored using Spearman’s correlation. All statistical analyses were performed using Microsoft Excel 2016 with the Analyse-it add-in (Microsoft, Redmond, WA, USA) and R version 4.3.1 (R Foundation for Statistical Computing, Vienna, Austria).

## 3. Results

### 3.1. Study Population

**Figure 1:**
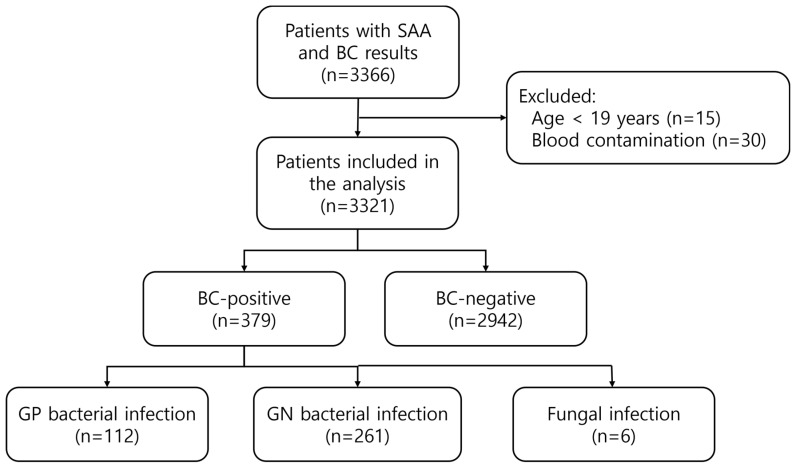
Flow diagram of patient selection and study population.

A total of 3366 patients underwent SAA and BC tests in the ED from January to December 2025. Among them, 15 patients aged <19 years and 30 patients with blood culture contamination were excluded. Therefore, the laboratory results of 3321 patients were included in the analysis. Among them, 1572 (47.3%) were female, and the median age was 71.3 (59.2–80.4) years. The median SAA level in the overall population was 86.8 (10.2–251.3) mg/L. Of the 3321 patients, 379 had positive BC results, corresponding to an overall BSI rate of 11.4%. Among them, 112 (29.6%) were identified as GP bacteria, 261 (68.9%) as GN bacteria, and 6 (1.6%) as fungi (Figure 1).

### 3.2. Association Between BC Results and SAA Levels

**Figure 2:**
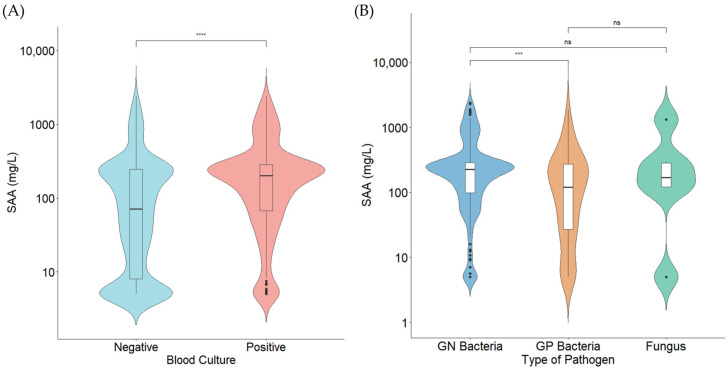
(**A**) SAA levels according to blood culture positivity. The median SAA levels were 202.0 (67.2–285.7) mg/L in the BC-positive group and 71.0 (8.0–245.5) mg/L in the BC-negative group (*p* < 0.001). (**B**) Comparison of SAA levels according to pathogen type. The median SAA levels were 225.6 (97.3–288.3) mg/L in the GN group, 179.7 (111.1–384.9) mg/L in the fungal group, and 119.5 (27.1–271.6) mg/L in the GP group. Pairwise comparisons following the Kruskal–Wallis test showed a significant difference between the GN and GP groups (*p* = 0.01), while no significant differences were observed among the other groups. The *y*-axis represents a log10 scale. *** *p* < 0.001, **** *p* < 0.0001, ns: not significant.

The median SAA levels were significantly higher in the BC-positive group than in the negative group (202.0 [67.2–285.7] mg/L vs. 71.0 [8.0–245.5] mg/L; *p* < 0.001). Among the BC-positive cases, significant differences in SAA levels were observed among the GP, GN, and fungal groups by the Kruskal–Wallis test (*p* = 0.022). In the post hoc pairwise analysis, the GN group showed significantly higher SAA levels compared with the GP group (*p* = 0.01). Specifically, SAA levels were the highest in the GN group (225.6 [97.3–288.3] mg/L), followed by the fungal group (179.7 [111.1–384.9] mg/L), and the GP group (119.5 [27.1–271.6] mg/L). These distributions and the differences among groups in SAA levels are described in Figure 2.

### 3.3. Correlation Between SAA and Other Laboratory Markers

Correlation analysis between SAA and other inflammatory markers, including CRP, PCT, WBC, and ANC, demonstrated that CRP had the strongest correlation with SAA (Spearman’s *r_s_* = 0.884). PCT exhibited the second-highest correlation with SAA, with a moderate correlation coefficient (Spearman’s *r_s_* = 0.576) (Table 1). Agreement between SAA and CRP showed a kappa value of 0.741 (95% CI: 0.714–0.767), with positive and negative agreement rates of 93.9% (95% CI: 92.9–94.8%) and 79.5% (95% CI: 76.6–82.0%). Agreement between SAA and PCT also showed a kappa value of 0.478 (95% CI: 0.443–0.514), with positive and negative agreement rates of 85.8% (95% CI: 84.4–87.1%) and 65.4% (95% CI: 61.8–68.9%).

### 3.4. Diagnostic Performance

**Figure 3:**
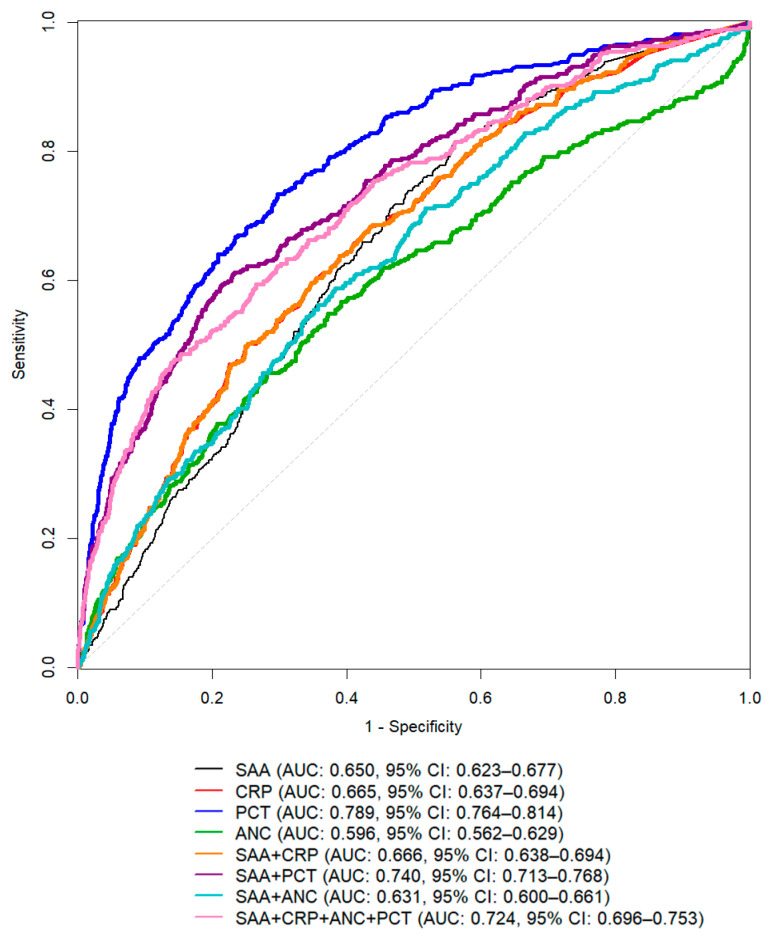
ROC curves of inflammatory markers alone and in combination in patients with bloodstream infection (BSI). The AUC for the diagnosis of BSI was highest for PCT (0.789), whereas SAA showed an AUC of 0.650. Combinations of SAA with CRP or ANC improved the AUC over individual markers but did not exceed that of PCT alone.

**Figure 4:**
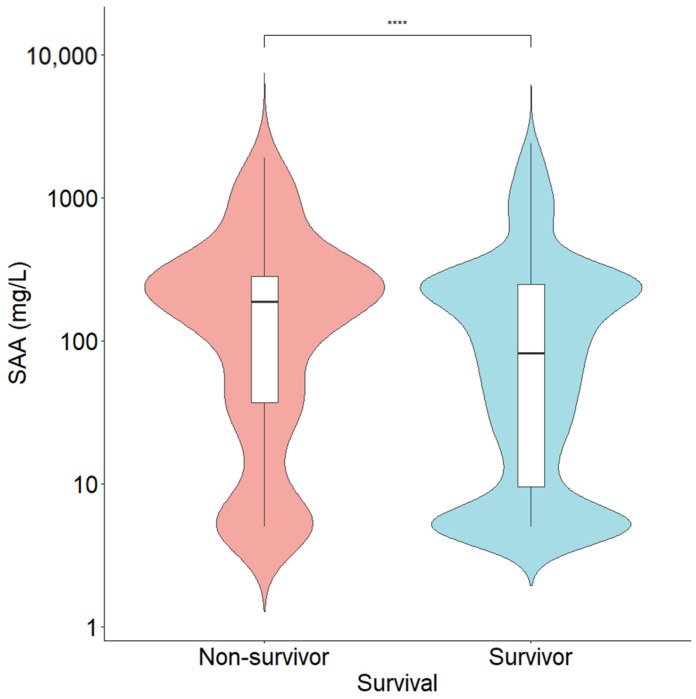
Comparison of SAA levels between non-survivors and survivors. The median SAA levels were 187.9 (36.2–384.3) mg/L in the non-survivor group and 81.6 (9.5–247.8) mg/L in the survivor group (*p* < 0.001). The *y*-axis represents a log10 scale. **** *p* < 0.0001.

To assess the diagnostic efficacy of SAA and other inflammatory markers, we compared the AUC values of BSI diagnoses using ROC curve analysis (Figure 3). Among the markers, the AUC for BSI diagnosis was the highest for PCT (0.789, 95% CI: 0.764–0.814), whereas SAA showed an AUC of 0.650 (95% CI: 0.623–0.677). CRP and ANC alone exhibited AUCs slightly lower than that of SAA, but the AUCs for CRP and ANC increased when combined with SAA in logistic models. For the diagnosis of BSI at the cutoff (10.0 mg/L), SAA demonstrated a sensitivity of 90.5% (95% CI: 87.1–93.1%) and a specificity of 26.9% (95% CI: 25.3–28.5%), with a positive predictive value of 13.8% (95% CI: 13.3–14.2%) and a negative predictive value (NPV) of 95.6% (95% CI: 94.1–96.8%) in our study population. Youden’s index was 0.174, and the OR was 3.504. When the optimal cutoff value was determined using the maximum Youden index, the calculated value was 86.8 mg/L. Among the 3321 patients included in this study, 248 (7.5%) died during hospitalization. The median SAA level was significantly higher in non-survivors (187.9 [36.2–384.3] mg/L) than in survivors (81.6 [9.5–247.8] mg/L) (*p* < 0.001) (Figure 4).

### 3.5. Prognostic Outcomes

**Figure 5:**
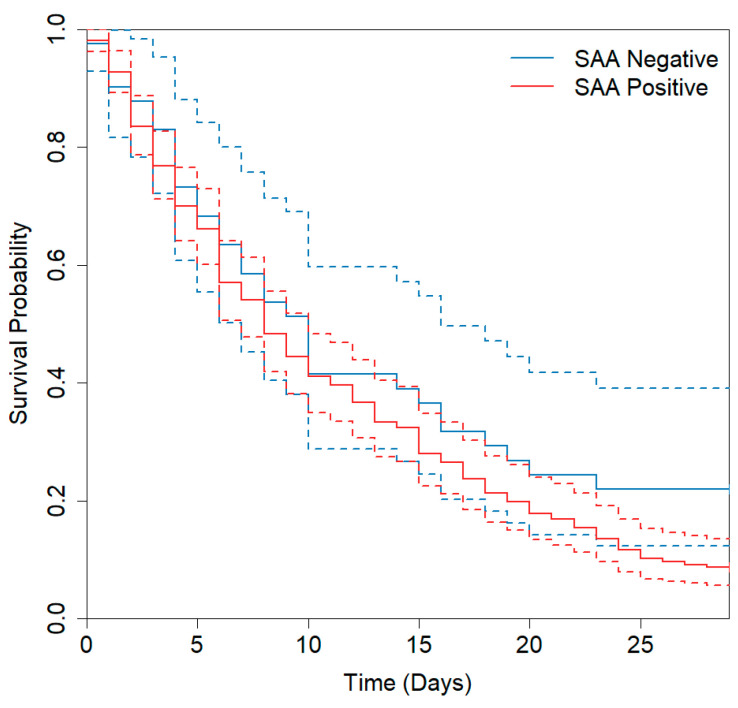
Kaplan–Meier survival curves according to SAA levels (cutoff: 10 mg/L). The dashed lines represent the 95% CI. The median survival time was 10 days (95% CI: 7–16 days) in the SAA-negative group and 8 days (95% CI: 7–10 days) in the SAA-positive group.

Kaplan–Meier survival analysis revealed that the median survival time was 10 days (95% CI: 7–16 days) in the SAA-negative group and 8 days (95% CI: 7–10 days) in the SAA-positive group (Figure 5). Among non-survivors, the length of hospital stay showed a negative trend with SAA levels; however, this trend was not statistically significant (Spearman’s *r_s_* = 0.004, 95% CI: −0.124–0.132, *p* = 0.569).

## 4. Discussion

In this large, single-center retrospective study of adult patients presenting to the ED, we investigated the diagnostic performance and prognostic value of SAA levels in relation to BSI and clinical outcomes. By including more than 3300 patients over a one-year period, our analysis provides robust real-world evidence regarding the behavior of SAA and its relationship with CRP, PCT, WBC, and ANC in an unselected ED population undergoing both SAA and BC testing. This study demonstrates that SAA levels were significantly higher in patients with positive BCs, particularly in those with GN BSI. In addition, SAA exhibited a strong correlation and agreement with CRP and showed high sensitivity, improving the diagnostic performance of CRP and ANC when used in combination.

SAA is one of the APRs synthesized rapidly by the liver in response to infection or inflammation and shows a particularly marked increase in bacterial infections, including BSIs. In GN bacterial infections, lipopolysaccharide (LPS) activates Toll-like receptor 4 (TLR4) signaling pathways, which serve as a pattern recognition receptor (PRR) [14]. This cascade leads to enhanced production of cytokines such as IL-6, IL-1β, and tumor necrosis factor-α (TNF-α) [25]. Consequently, this inflammatory response results in a rapid and marked increase in circulating SAA levels via its synthesis in hepatocytes [26,27]. While other inflammatory markers increase during GN bacterial infections, SAA increases more rapidly because of its unique induction mechanism. The expression of SAA is rapidly induced through the activation of transcription factors such as nuclear factor-kappa B (NF-κB) and signal transducer and activator of transcription 3 (STAT3) [1,28,29]. This was also reported in another study that evaluated SAA as an early diagnostic marker of late-onset sepsis in preterm infants, with SAA levels significantly higher in GN compared to GP sepsis [15]. Our observation that SAA levels were significantly higher in patients with positive blood cultures, with the most notable elevation observed specifically in patients with GN bacterial infections, is consistent with these previous insights.

SAA showed the strongest positive correlation with CRP in the correlation and agreement analyses with other inflammatory markers, which aligns with prior findings from ED and outpatient cohorts demonstrating parallel kinetics of these APRs across a wide range of inflammatory states [19,30]. However, subtle differences in the kinetics and magnitude of SAA remain clinically relevant. Several studies have reported that SAA rises and declines more rapidly than CRP, potentially making it a more suitable marker for early detection and short-interval monitoring of treatment response [4,31,32,33,34]. Furthermore, SAA may exhibit a broader dynamic range than CRP in certain infections, such as neonatal sepsis and postoperative peritonitis, where SAA outperformed CRP and PCT in some diagnostic models [15,16,35,36,37]. Our finding that SAA improves the AUC of CRP and ANC when used in combination suggests that SAA captures aspects of the inflammatory response that are not fully represented by these markers and supports the use of multimarker strategies in ED triage.

The AUC analysis revealed that PCT alone had the largest AUC. The combination of SAA and PCT yielded the second-largest AUC, outperforming other individual markers or combinations. SAA demonstrated high sensitivity (90.5%) and NPV (95.6%) at the institutional cutoff, albeit with limited specificity (26.9%). Although PCT may provide higher sensitivity and better discrimination for sepsis compared with CRP and SAA, PCT testing may be more expensive or less readily available than CRP and SAA in some settings. In this regard, SAA could serve as an adjunctive biomarker that complements PCT for ruling out BSI in low-risk patients or in settings where PCT testing is unavailable due to resource constraints or platform limitations.

The institutional cutoff of 10 mg/L was established based on reference intervals according to manufacturer recommendations. In contrast, the optimal cutoff of 86.8 mg/L, derived from Youden’s index, provides improved specificity at the cost of reduced sensitivity and may be more appropriate for diagnostic purposes in higher-risk patients.

Mortality analyses showed that non-survivors had significantly higher baseline SAA levels than survivors, and SAA-positive patients had shorter median survival times. These results are in accord with prior studies on COVID-19 and other infections, which reported that elevated SAA levels were associated with higher scores on severity scales, more extensive organ involvement, and worse outcomes [4,38,39,40,41]. The association between higher SAA concentrations and poor prognosis may be partly explained by its role in amplifying cytokine responses and immune cell activation. Nevertheless, elevated SAA does not necessarily reflect disease severity alone, as it may also be increased due to chronic comorbid conditions or generalized tissue injury. Accordingly, SAA is likely to be most useful as an adjunctive prognostic biomarker integrated with established clinical severity scores and organ dysfunction indicators, rather than as a standalone predictor.

This study has several limitations. First, we were unable to evaluate the temporal changes in SAA levels or the precise timing of SAA measurement from symptom onset. As SAA is a rapidly responsive inflammatory marker, variability in the timing of measurement may have affected its diagnostic performance, and the inclusion of symptom onset timing or serial SAA measurements could potentially influence the ROC curve results. Second, patients with BSI were classified only according to the identified pathogens. However, further stratification based on specific diagnoses or detailed clinical presentations was not performed, which may have introduced heterogeneity and limited our ability to detect more granular associations between SAA and particular infection sources. Additionally, patients with true BSI who had false-negative BCs due to the inherent sensitivity limitations of this method would have been misclassified as non-BSI, potentially underestimating the true diagnostic performance of SAA. Third, information on comorbidities such as chronic inflammatory diseases, malignancy, and prior antimicrobial therapy was not systematically collected; these conditions are known to elevate SAA and may have contributed to the low specificity. Therefore, the survival analysis was based on unadjusted Kaplan–Meier curves without controlling for confounders such as comorbidities, infection sources, or infection severity, indicating that the independent prognostic value of SAA could not be established. Finally, as this study was performed retrospectively, based on routine clinical data, laboratory results for other APRs such as ESR, fibrinogen, ferritin, IL-6, and haptoglobin were not consistently available, precluding more extensive multimarker analysis.

## 5. Conclusions

In conclusion, our study suggests that SAA, measured at ED presentation, is significantly associated with BSI and in-hospital mortality and provides high sensitivity for BSI detection in adult patients. While SAA alone has moderate discriminative power, the combined use of SAA with complementary biomarkers may contribute to early risk stratification, though its independent prognostic role requires validation in future prospective studies. Future prospective multicenter studies across different patient populations are warranted to validate the optimal SAA cutoffs derived in this study (86.8 mg/L), clarify its relationship with symptom onset and treatment response, and determine strategies for improving patient outcomes in the ED using SAA.

## Figures and Tables

**Table 1:** Correlation between SAA and other laboratory tests in all patients.

	Spearman’s *r_s_* (95% CI)	*p*
CRP	0.884 (0.876 to 0.891)	<0.001
PCT	0.576 (0.552 to 0.599)	<0.001
WBC	0.231 (0.197 to 0.264)	<0.001
ANC	0.300 (0.267 to 0.331)	<0.001

Abbreviations: SAA, serum amyloid a; CRP, C-reactive protein; PCT, procalcitonin; WBC, white blood cell; ANC, absolute neutrophil count.

## Data Availability

The datasets used and analyzed during the current study are available from the corresponding author upon reasonable request.
